# Combining mechanistic modeling with machine learning as a strategy to predict inflammatory bowel disease clinical scores

**DOI:** 10.3389/fphar.2025.1479666

**Published:** 2025-02-25

**Authors:** Jaehee V. Shim, Markus Rehberg, Britta Wagenhuber, Piet H. van der Graaf, Douglas W. Chung

**Affiliations:** ^1^ Certara Applied BioSimulation, Sheffield, United Kingdom; ^2^ Sanofi R&D, Translational Disease Modeling, Frankfurt amMain, Germany; ^3^ Division of Systems Pharmacology and Pharmacy, Leiden Academic Centre for Drug Research, Leiden University, Leiden, Netherlands

**Keywords:** inflammatory bowel disease (IBD), machine learning, mayo score prediction, endoscopic score prediction, quantitative systems pharmacology (QSP) model, CDAI score prediction

## Abstract

Disease activity scores are efficacy endpoints in clinical trials of inflammatory bowel disease (IBD) therapies. Crohn’s disease activity index (CDAI), Mayo endoscopic score (MES) and Mayo score are frequently used in clinical trials. They rely on either the physician’s observation of the inflammatory state of the patient’s gastrointestinal tissue alone or combined with the patient’s subjective evaluation of general wellbeing. Given the importance of these scores in evaluating the efficacy of drug treatment and disease severity, there has been interest in developing a computational approach to reliably predict these scores. A promising approach is using mechanistic models such as quantitative systems pharmacology (QSP) which simulate the mechanisms of the disease and its modulation by the drug pharmacology. However, extending QSP model simulations to clinical score predictions has been challenging due to the limited availability of gut biopsy measurements and the subjective nature of some of the evaluation criteria for these scores that cannot be described using mechanistic relationships. In this perspective, we examine details of IBD disease activity scores and current progress in building predictive models for these scores (such as biomarkers for disease activity). Then, we propose a method to leverage simulated markers of inflammation from a QSP model to predict IBD clinical scores using a machine learning algorithm. We will demonstrate how this combined approach can be used to (1) explore mechanistic insights underlying clinical observations; and (2) simulate novel therapeutic strategies that could potentially improve clinical outcomes.

## Introduction

Inflammatory bowel disease (IBD) is a chronic inflammatory gut disease prevalent in the United States with approximately 1.6 million residents affected and over two million are estimated to be suffering in Europe (Gajendran et al., 2018; Ramos and Papadakis, 2019). IBD is classified into two subtypes (1) Crohn’s disease (CD) which can affect anywhere from the mouth to the perianal area and (2) ulcerative colitis (UC) primarily affects the large intestine (Gajendran et al., 2018; Gajendran et al., 2019; Rogers et al., 2021a). Both types of IBD can present with complications such as fistulae and strictures which may require surgery (Kim et al., 2013; Kishi et al., 2022; Rogers et al., 2021a; Venkatapurapu et al., 2022).

The pathogenesis of IBD involves environmental factors that can influence the changes in the microbiome and genetic factors that increase the susceptibility to gut inflammation. The disease mechanism involves a pathogenic microbiome entering through a weakened intestinal barrier which then leads to a dysregulated mucosal immune response ending in relapsing-remitting gut inflammation (Ahluwalia et al., 2018; Ramos and Papadakis, 2019; Sartor, 2006).

Many approved IBD therapeutics target immunological dysregulation to reduce gut inflammation and ameliorate symptoms. Immunomodulatory drugs that have been shown to clinically improve IBD symptoms include (1) tumor necrosis factor (TNF) antibodies (infliximab, adalimumab, golimumab), (2) interleukin (IL) 12/23 antibodies (ustekinumab, mirikizumab, guselkumab, risankizumab, brazikumab), (3) integrin antibodies (carotegrast, vedolizumab), and (4) Janus kinase (JAK) inhibitors (tofacitinib, filgotinib, upadacitinib) (Kobayashi and Hibi, 2023; Rogers et al., 2021a).

To better understand the disease biology and optimize therapeutic strategies, there has been significant interest in developing quantitative systems pharmacology (QSP) models that can capture the interactions between different immune cells, cytokines, interleukins, epithelial barrier, and gut microbiome (Balbas-Martinez et al., 2018; Fendt et al., 2024; Lo et al., 2013; Pinton, 2022; Pinton, 2023; Rogers et al., 2021b; Rogers et al., 2021a; Stübler et al., 2023; Venkatapurapu et al., 2022; Wendelsdorf et al., 2010; Whittaker et al., 2024). Because model connections are calibrated to available data, typically the model output is in the form of clinically measurable biomarkers such as fecal calprotectin (FCP) and serum c-reactive protein (CRP) (Rogers et al., 2021a; Rogers et al., 2021b; Venkatapurapu et al., 2022). However, in clinical trials, clinical scores are preferred metrics over biomarkers such as FCP and serum CRP because, while biomarkers are useful indicators to infer endoscopically active IBD, their levels do not necessarily correlate with disease activity level (Falvey et al., 2015; Wagatsuma et al., 2021). To reliably predict IBD clinical scores, there needs to be sufficient training data relating gut inflammation to measurable clinical markers and clinical scores. The IBD QSP model can generate simulated data on gut immunocytes and cytokine levels overcoming the limited quantity of patient data. The simulated patient data can be utilized to train a machine-learning model to describe the relationships between gut inflammatory markers and IBD clinical scores.

Here, we present a computational strategy to reliably generate comprehensive clinical score predictions for both UC and CD using gut-level biomarker simulations with a published IBD QSP model (Rogers et al., 2021a; Rogers et al., 2021b). We will showcase how such an integrated model can be used to explore (1) potential mechanistic differences behind responders versus non-responders of anti-TNFα and anti-IL-23 combination therapy in UC patients (inspired by a recent study by Feagan et al. (2023)); and (2) if the same dual combination would be beneficial for the other IBD subtype, CD.

## IBD clinical scores

Clinical scores are utilized in clinical practice and in drug development to assess disease severity and can be categorized into (1) overall activity score which is a comprehensive measurement of disease severity, and (2) endoscopic score which is solely based on physical symptoms observed during endoscopy. For each subtype of IBD, there are various standardized scores for both overall and endoscopic assessment.

For UC, the most popular index for overall disease activity is the Mayo score (partial Mayo included). For clinical trials conducted from 2013 to 2017, about half of the trials (49.5%) utilized the Mayo score as clinical index to evaluate therapeutic responses (Kishi et al., 2022). Related indices include the Clinical Activity Index (CAI), Disease Activity Index (DAI), and Simple Clinical Colitis Activity Index (SCCAI). The most popular choice for the endoscopic index for UC was the Mayo Endoscopic Score (MES), which was employed in 69.0% of the clinical trials conducted from 2013 to 2017 (Kishi et al., 2022). Additional commonly used endoscopic indices are the Sutherland Endoscopic Sub-score and Ulcerative Colitis Endoscopic Index of Severity (UCEIS).

In CD, the most used disease activity index in clinical trials for overall assessment was the Crohn’s Disease Activity Index (CDAI). Alternatively, the Harvey-Bradshaw Index (simple CDAI) is also utilized. For the endoscopic evaluation of CD, the CD Endoscopic Index of Severity (CDEIS) has been noted as the gold standard. Other options that have been utilized in place of the CDEIS are the Simple Endoscopic Score for Crohn’s disease (SES-CD) and the Rutgeert Score (Kim, 2022; Kishi et al., 2022).

The most popular clinical indices in each category for CD (CDAI for overall, CDEIS for endoscopic score) and UC (Mayo for overall, MES for endoscopic score) are reviewed in more detail.

## CDAI

The CDAI was first introduced in 1976 as a part of the National Cooperative Crohn’s Disease Study and has since been considered the gold standard for evaluating CD (Kishi et al., 2022; Liu and Lichtenstein, 2012). To assign a CDAI score, there are eight items evaluated over 7 days (Freeman, 2008; Kishi et al., 2022; Liu and Lichtenstein, 2012). Some items are highly subjective such as abdominal pain score in 1 week and general wellbeing. Other factors considered are the number of liquid or very soft stools, the sum of physical findings per week (fever, anal disease, mucocutaneous lesions, arthritis, external fistula), antidiarrheal use, abdominal mass, and low hematocrit count. Each of the evaluation criteria is multiplied by a weighting factor and summed to derive the score (Freeman, 2008; Kishi et al., 2022). The CDAI scores below 150 are interpreted as no disease activity, 150–220 as mild, 220–450 as moderate, and >450 as severe (Nakamura et al., 2018; Shinzaki et al., 2021). For the clinical trials conducted from 2009 to 2017, the CDAI was reported to be the most frequently used index in over 50% of trials (Kishi et al., 2022).

## CDEIS

The CDEIS was developed in 1989 by the Groupe d’Etudes Therapeutiques des affections Inflammatoires du tube Digestif. Items considered for the CDEIS scoring include the presence of superficial or deep ulcerations and the percentage of ulcerated surface. Scores can range from 0 to 44 and can be interpreted as healed if the score is 0–3, mild disease 4, moderate disease 5–15, and severe disease > 15 (Kim, 2022). The CDEIS has been the most popular choice in clinical trials for 2009–2012 while the SES-CD has been preferred in more recent trials (Kim, 2022; Kishi et al., 2022).

## Mayo

The Mayo score was published in 1987 by Schroeder et al. in a study to evaluate the therapeutic effects of coated oral 5-aminosalicylic acid for UC (Kishi et al., 2022; Schroeder et al., 1987). Since then, the Mayo score has been the most utilized index in clinical trials, but its validity has not been examined thoroughly (Kishi et al., 2022). Scoring is based on four items which include stool frequency, rectal bleeding, mucosal appearance at endoscopy and physician rating of disease activity. Each item is rated between 0 and 3 and the final score is derived by adding up the ratings from each category (J. D. Lewis et al., 2008). The Mayo score is interpreted as remission if the score is 0–2, mild if 3-5, moderately active if 6–10 and severely active if 11–12 (CADTH, 2016).

## MES

The MES was developed as a component for the Mayo score by Schroeder et al. in the 1987 study (Schroeder et al., 1987). It is evaluated in four points, ranging from 0–3, where the MES score of 0 represents normal and 3 represents an ulcerated state (Kim, 2022; Kishi et al., 2022). The descriptors utilized in the MES assessment include erythema, vascularity, friability, bleeding, erosions, and ulcerations (Kim, 2022). While the MES is the most popular endoscopic index for recent clinical trials, its validity has not been sufficiently evaluated (Kim, 2022; Kishi et al., 2022).

## Challenges of and progress toward extending the QSP model to predict IBD clinical scores

Mechanistic modeling of IBD has been valuable in understanding the disease pathophysiology, exploring therapeutic targets, and optimizing treatment strategies (Balbas-Martinez et al., 2018; Kilian et al., 2024; Lo et al., 2013; Pinton, 2022; Pinton, 2023; Rogers et al., 2021a; Stübler et al., 2023; Venkatapurapu et al., 2022; Wendelsdorf et al., 2010; Yu et al., 2024). Published IBD models include (1) microbiome and epithelial barrier dysfunction, (2) immunological dysregulation in lamina propria and lymph nodes involving innate immune cells (neutrophils, macrophages, dendritic cells, natural killer T cells) and adaptive immune cells (B cells and T cells) (Abraham and Cho, 2009; Cai et al., 2021; Kilian et al., 2024; Saez et al., 2023; Stübler et al., 2023; Yu et al., 2024). Because many of the approved drugs target distinct immunological pathways, there is increasing interest in finding synergistic combinations of existing drugs to optimize therapeutic outcomes (Dai et al., 2023; Feagan et al., 2023; Pinton, 2022; Wetwittayakhlang and Lakatos, 2024). For instance, a recent study by Feagan et al. showed that dual targeting of TNFα and IL-23 almost doubled the clinical remission rate compared to single-targeting therapies (Feagan et al., 2023). Other effective combinations include ustekinumab and vedolizumab (Dawoud et al., 2022), infliximab and azathioprine (Sultan et al., 2017), as well as adalimumab and vedolizumab (Goessens et al., 2021).

Identifying optimal dual targeting strategies has become the main application of QSP IBD modeling. However, many of the published models (Abraham and Cho, 2009; Cai et al., 2021; Kilian et al., 2024; Saez et al., 2023; Stübler et al., 2023; Yu et al., 2024) lack the extension to predict clinical scores. Having the capacity to simultaneously simulate changes in gut-level dynamics of cytokines and immune cells along with clinical scores enables a mechanistic understanding of treatment response by closely matching virtual populations with the biomarker and endpoint response in real-life clinical trials.

Although there is great interest in predicting clinical efficacy, extending IBD models to clinical scores has been a challenging task. As previously discussed, these scores include highly subjective criteria such as “general wellbeing” and “physician’s rating of disease” proving challenging to “mechanistically link” with disease biology. Even endoscopic scores have been reported to vary between physicians (Kishi et al., 2022). Furthermore, there is limited availability of individual-level patient biopsy data that contain both gut-level biomarkers and clinical score measurements.

To overcome these difficulties, a recent study (Venkatapurapu et al., 2022) has employed a hybrid mechanistic-statistical platform that simultaneously simulates Crohn’s disease progression and incorporates a simple decision tree-based classifier to generate a prediction for SES-CD scores using features such as lesions, biomarkers, and the duration of CD. This platform takes in the patient information, disease profile and treatment history as the input for the responder classifier and generates a long-term response which is then used to identify a matching virtual patient from a virtual population library generated using mechanistic disease modeling. The authors successfully demonstrated that this hybrid method can generate a time series of SES-CD scores along with biomarkers, gut-level cytokines, and immune cell population changes.

This approach of combining a classification algorithm with a mechanistic model is a promising way to extend the prediction because disease activity indices are typically interpreted in categories such as no activity/remission, mild, moderate, and severe. Furthermore, the machine learning component enables learning from literature-reported correlations between biomarkers and clinical scores while the mechanistic model requires a specific data format to be used for calibration. For instance, transcription factors and cytokine levels from isolated tissue biopsies of active IBD patients and healthy controls can be used to correlate with disease severity as potential predictors to be trained and cross-validated in the machine learning model (Li et al., 2017; Olsen et al., 2011).

Additionally, the most notable benefit of the combined approach is that it can be applied to already published models that have been validated. For instance, Rogers et al. recently published a comprehensive QSP IBD model that can simulate both CD and UC conditions (Rogers et al., 2021a; Rogers et al., 2021b). The model includes important disease biology in the gut and blood involving T cells, macrophages, dendritic cells, and neutrophils along with cytokine release. The model also generates clinical biomarker simulations such as CRP and FCP which are often reported with clinical scores. The authors followed up with additional virtual population analyses by simulating the treatment effects of four different therapeutic targets in CD. They also explored a dual therapy option, anti-TNFα, and anti-IL-12p40, as a potential treatment option, and the simulation predicted that a combination strategy would improve the response compared to mono-therapies (Rogers et al., 2021b). The model code is publicly available to be downloaded in the supporting information section of the Rogers et al. publication.

Given the demonstrated capabilities of the Rogers et al. model, we linked it to a machine learning algorithm to predict clinical scores for both subtypes of IBD and performed a proof-of-principle virtual population analysis.

## Clinical score predictions using a combination of a large-scale QSP model and statistical learning approach and example applications

For implementing a clinical score prediction model, we selected the Mayo score (with MES, which is a part of Mayo) for UC and CDAI for CD as output, since they are the most popular metrics in clinical trials. The model was designed to predict the “range of the scores,” defined by clinicians to categorize the severity of the disease. To ensure interpretability, we employed multinomial logistic regression (MLR) in MathWorks (2024), a simple machine-learning classification algorithm, to generate predictions of categorical ranges of clinical scores. Feature selection for the MLR model was guided by the literature evidence between gut biomarkers and the relevant clinical scores (Table 1).

**TABLE 1:** Gut biomarkers reported to correlate with clinical scores of IBD.

Correlations	Th1	Th2	Th17	Treg	NK/NKT	Mac (M1/M2)	DC	Neutrophil	References
UC endoscopic/clinical activity			IL-17, IL-21, IL-22, TGFβ	% CD25^hi^ FOXP3+ (T cells), TGFβ	IL-17, IL-21, IL-22, TGFβ	IL-8, TGFβ		IL-8	Holmén et al. (2006), Iboshi et al. (2017), Jiang et al. (2014), Zahn et al. (2009)
UCDAI/Mayo	TNFα, IFNγ	%CD4+IL13+FOXP3+ (Th2/Treg)	IL-6	%CD4+IL13+FOXP3+ (Th2/Treg)	IFNγ	TNFα, IL-6, IL-23	IL-6, IL-23, IFNγ	IL-6, IL-23	Allegretti et al. (2023), Li et al. (2017), Olsen et al. (2007), 2011
CDAI	TNFα		IL-6, IL-17, TGFβ	TGFβ	IL-17, TGFβ	TNFα, IL-6, TGFβ, IL-8	IL-6	IL-6, IL-8	Olsen et al. (2007), 2011; Stallmach et al. (2004)

**FIGURE 1:**
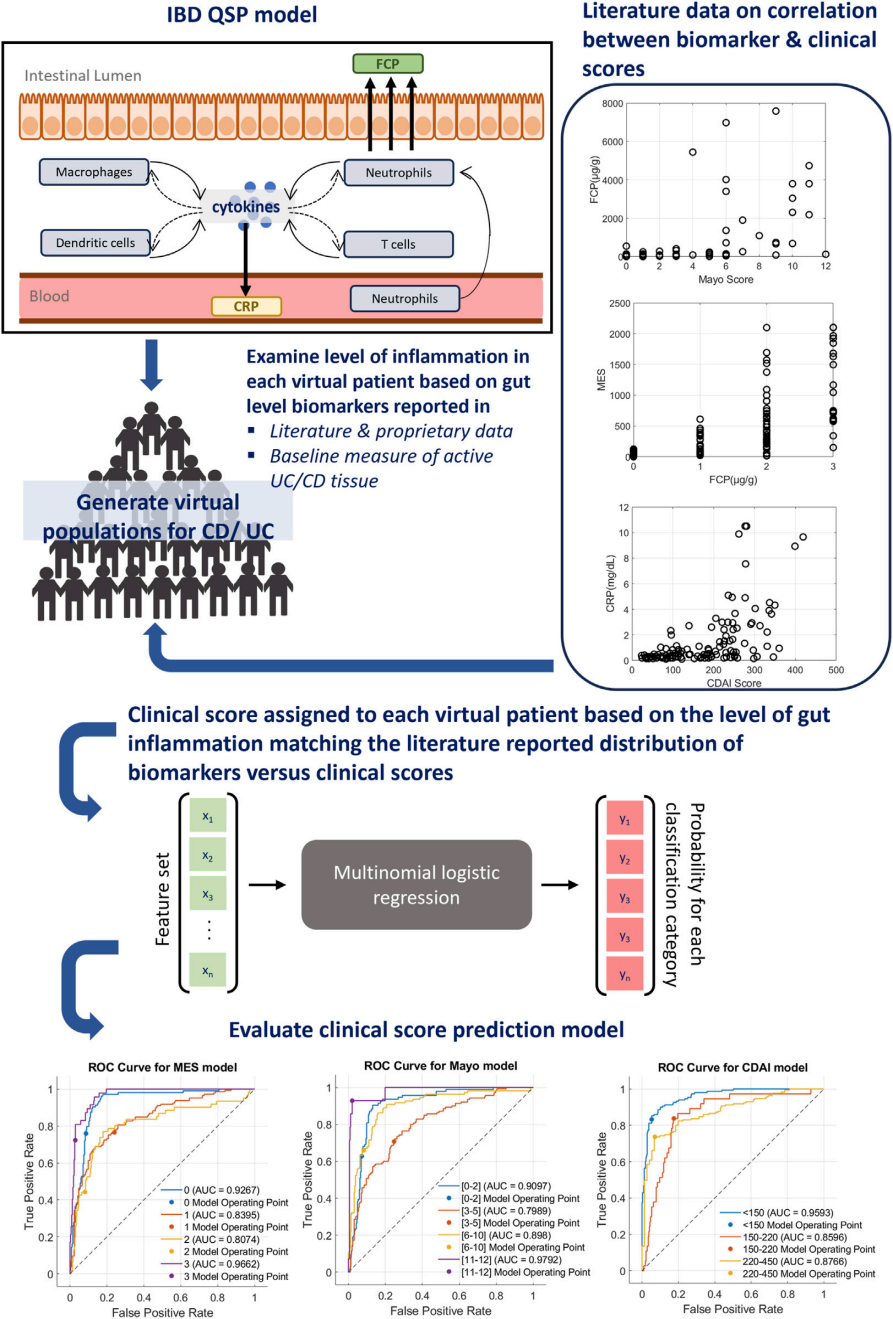
Overview of IBD score prediction platform. A clinical prediction algorithm has been integrated with a published mechanistic model of IBD that can simulate both CD and UC. For the interpretability of features, a simple classification algorithm, multinomial logistic regression (MLR), has been selected to build clinical prediction extension. First, the level of inflammation in each virtual patient was computed based on the simulation of relevant tissue biomarkers and serum CRP or FCP. Next, a relevant clinical score was assigned to each virtual patient using the literature reported relationship of clinical scores and CRP or FCP. The actual clinical score distribution data utilized for this process are shown on the right. Using this population, the MLR model was trained to generate clinical score predictions and the performance was evaluated using metrics such as the ROC curve (bottom), sensitivity and specificity measures (Supplementary Tables S2-S4).

To generate training data, we developed an algorithm that assigns appropriate clinical scores to the published virtual population of the Rogers et al. model based on (1) literature data of IBD score distribution (Kawashima et al., 2016; Nakamura et al., 2018; Shinzaki et al., 2021) in relation to either FCP or CRP; and (2) simulated levels of tissue biomarkers that had both strong correlations with IBD scores (Holmén et al., 2006) (Table 1) and known mechanistic links with IBD pathology in the literature (Langer et al., 2019). This algorithm computes an overall inflammatory score based on the QSP model-generated steady-state values of relevant tissue biomarkers and serum CRP and FCP levels to match published distributions comparing IBD clinical scores with serum CRP or FCP of individual patients (Kawashima et al., 2016; Nakamura et al., 2018; Shinzaki et al., 2021). Once inflammatory scores were assigned to UC and CD virtual populations, they were used to train the MLR model for the relevant clinical score. An overview of this computational pipeline is described in Figure 1.

The performances of trained models were evaluated using Receiver Operating Characteristic (ROC) curves and sensitivity/specificity metrics (Figure 1; Supplementary Tables S2-S4). ROC curves indicate that the trained models are operating at a consistent level across all classes with moderate to strong predictability specific to the class. Additionally, sensitivity and specificity measures (Supplementary Tables S2-S4) for each MLR model further highlight the strength of the model performance with the average sensitivity/specificity of the MES model at 0.67/0.84, Mayo model at 0.73/0.88, and CDAI model at 0.80/0.89.

Next, exploratory analyses were performed using anti-TNFα (adalimumab), anti-IL-23 (mirikizumab), and the combination. These targets were selected based on the recent study by Feagan et al. (2023) where simultaneous inhibition of TNFα and IL-23 led to significant improvement of therapeutic response in UC patients. The clinical trial data for adalimumab (ECCO, 2024; Puri et al., 2017; Shinzaki et al., 2021) and mirikizumab (Sandborn et al., 2020; Sands et al., 2022) were used to calibrate the model further. Once the clinical score prediction algorithm was built and calibrated, additional analyses were performed to demonstrate whether this hybrid approach can be used to explore (1) mechanistic differences between clinical responders versus non-responders of dual targeting in the UC virtual population; and (2) whether this combination could also be beneficial for treating CD.

**FIGURE 2:**
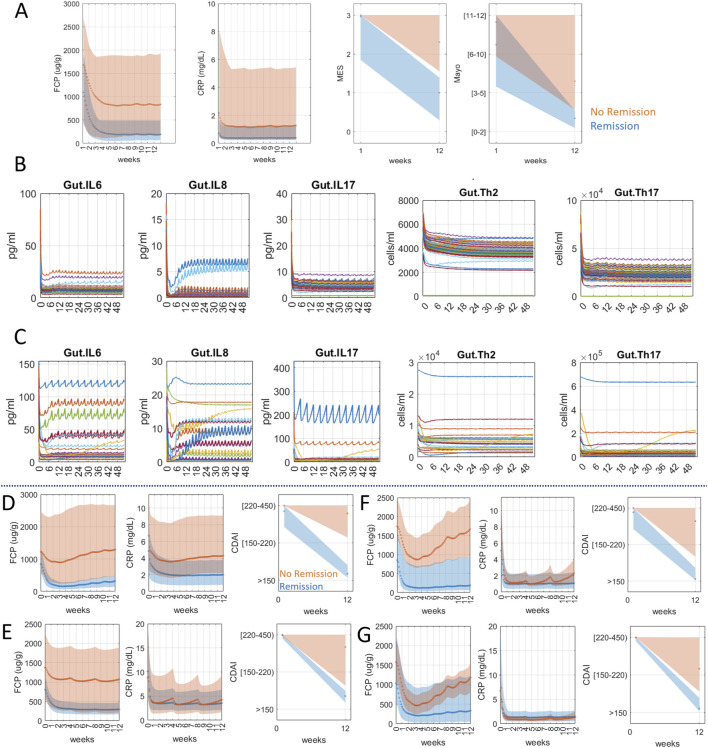
Anti-TNFα and anti-IL23 targeting simulations for UC **(A–C)** and CD **(D–G)** virtual population. **(A)** UC virtual population matching the response data of dual targeting of anti-TNFα and anti-IL23 in the VEGA trial (49.3% endoscopic healing and 36.6% clinical remission, MES ≤ 1 and Mayo ≤ 2, at week 12). Orange shaded area represents the population that reached remission in 12 weeks (dark orange dots in CRP/FCP plots represent mean values). The blue-shaded area represents the population that did not reach remission in 12 weeks (dark blue dots in CRP/FCP plots represent mean values). **(B, C)** Mechanistic plots of UC virtual population treated with combination therapy. Each line represents an individual virtual patient. The x-axis is on the scale of weeks. Simulation shows dual therapy was not as effective in bringing down IL6, IL8, IL17, Th2 and Th17 in non-responders **(C)** as in responders **(B)**. **(D)** CD virtual population matching adalimumab clinical trial data on baseline CDAI (271 ± 56) and treatment response (remission 67% at 12 weeks) based on Shinzaki et al. (2021). **(E)** CD virtual population matching mirikizumab clinical trial data on baseline CDAI (298 ± 103.7) and treatment response (remission 40.6% at 12 weeks) based on Sands et al. (2022). Both CD virtual populations in **(D)** and **(E)** were selected using reported CRP, FCP levels in the trial as well as the reported CDAI distribution at week 12. **(F)** Virtual CD population matching adalimumab trial data were subjected to hypothetical combination therapy (adalimumab and mirikizumab). The model simulation showed 86% remission at week 12, a significant improvement from the adalimumab trial population (66% remission) **(G)** Virtual CD population matching mirikizumab trial data were simulated for combination therapy and the result showed 81% remission at week 12, a notable jump from 41%.

For the first part of the exploratory analysis, we generated a UC virtual population (Figure 2A) that matched the patient data published in the VEGA trial (Feagan et al., 2023). This trial showed, that in 12 weeks, 37% of patients treated with combination reached remission while single targeting only reached 21% (anti-IL23, Guselkumab) and 22% (anti-TNFα, Golimumab), respectively. Elaborating on this effort, using mechanistic modeling, we sought to examine the mechanistic differences between the responder population who reached remission in 12 weeks versus the non-responder population (Figures 2B,C). Simulation shows dual targeting was able to significantly decrease IL6, IL8, and IL17 and reduce cell populations of Th2 and Th17 better in responders (Figure 2B) than non-responders (Figure 2C). Additional responder versus non-responder analysis can be performed to identify potential biomarkers of response before treatment.

Next, we evaluated whether anti-TNFα and anti-IL-23 combination could also benefit CD, given the overlapping biology with UC. Because this combination has not been evaluated for CD, we generated two separate CD virtual populations that match the published clinical data for each therapy, adalimumab (Shinzaki et al., 2021) (Figure 2D) and mirikizumab (Sands et al., 2022) (Figure 2E) and evaluated their combination. The results show that, in both virtual populations, combination therapy is predicted to significantly improve the clinical outcome (Figures 2F,G). Simulation shows, for evaluating 12 weeks remission rates, adalimumab and mirikizumab alone were 66% and 41% respectively whereas combination therapy in the same virtual population for both therapies led to 86% and 81%. This is an intriguing result that encourages further testing in the clinic. Thus, a model that can simulate clinical scores enables the exploration of novel drug combinations while providing a mechanistic explanation.

The simulation results show the potential of combining machine learning with mechanistic modeling to predict clinical scores and disease activity indices using publicly available models and data. Furthermore, matching the clinical response rates in UC simulations verifies the clinical score calculation and emphasizes its more general and robust use.

## Future directions

A key challenge in validating a predictor of an IBD clinical score is the lack of publicly available individual patient gut biopsy measurements of key cytokines and immune cell activity paired with the actual clinical score. To accommodate for this limitation, we first simulated a validated virtual population from a published model, then selected the virtual patients that matched the FCP or CRP levels of real individual patient data and assigned the associated clinical scores. While the purpose of this manuscript is to showcase a proof-of-principle analysis that would enable extending QSP model prediction to a clinical disease score, an in-depth validation using patient-level biopsy data in the future would strengthen this approach. Another validation step can be to predict various clinical studies with different treatments to validate the MES response predicted by the model. If the prediction achieves enough confidence an analysis of patient endotypes may give important insights to choose the right drug for the right patient.

Another ongoing effort is the CODEX database (Certara, 2024) which is a collection of biomarkers and clinical scores from published literature sources formatted for validating mechanistic models. Future studies will only enrich the database that can be utilized to bolster the model prediction. On top of these challenges, most clinical scores have empirical and subjective elements that require regression to relate to a mechanistic model. Our QSP model-based approach can leverage population-level distributions of key biomarkers and clinical scores at baseline, induction, and maintenance periods to enrich the UC and CD virtual population improving the accuracy and precision of the QSP model outputs and clinical scores.

Although IBD clinical scores hold the most practical value, significant efforts distinguishing IBD subtypes based on gene expression profiles have been measured at disease onset or the initiation of therapy (Barber et al., 2016). Indeed, integrating omics data sources to inform mechanistic models have been demonstrated in other fields such as oncology and cardiotoxicity (Lazarou et al., 2020; Shim et al., 2023).

Differential gene expression data could serve as inputs to a clinical score predictor; however, such attempts are challenging to validate. Additionally, genetic markers are not regularly measured and may not be robust predictors of response. In addition, such markers are hard to model mechanistically due to the lack of understanding how they link to cell or tissue-level pathophysiology. There has been limited success in other indications such as rheumatoid arthritis (Dennis et al., 2014; Guan et al., 2019; Lewis et al., 2019). In future studies, gene biomarkers may be incorporated into QSP models, alongside cytokine data, to enhance diagnostic or prognostic capabilities.

## Conclusion

We presented the contemporary challenges of adding a clinical score predictor to a mechanistic model of IBD illustrating how a combined QSP/machine learning approach could provide a way to overcome those challenges. A recent hybrid approach combined a simple decision tree algorithm with a QSP model to generate SES-CD scores, an endoscopic score of CD, demonstrating the potential of combining machine learning with mechanistic models (Venkatapurapu et al., 2022). Our work expands the clinical prediction for both UC and CD combining a QSP model of IBD with a MLR algorithm to generate relevant clinical scores with interpretable and literature-supported features. Efforts are underway to leverage additional clinical trial data from our CODEX (Certara, 2024) clinical outcomes database to enrich training and testing data sets and improve the accuracy of this approach.

## Data Availability

The datasets presented in this study can be found in online repositories. The names of the repository/repositories and accession number(s) can be found in the article/Supplementary Material.
