# Supplementary material for: Combining mechanistic modeling with machine learning as a strategy to predict inflammatory bowel disease clinical scores
Source: Front Pharmacol. 2025 Feb 25;16:1479666. doi: 10.3389/fphar.2025.1479666 (PMC11893853; doi:10.3389/fphar.2025.1479666)
Supplement: Supplementary file 1 [file Table1.docx]

Supplementary Material

**Combining mechanistic modeling with machine learning as a strategy to predict inflammatory bowel disease clinical scores.**

Jaehee V. Shim^1^, Markus Rehberg^3^, Britta Wagenhuber^3^, Piet H. van der Graaf^1,2^, Douglas Chung^*1^

^1^Certara Applied BioSimulation, Sheffield, UK

^2^Leiden Academic Centre for Drug Research, Division of Systems Pharmacology and Pharmacy, Leiden University, The Netherlands

^3^Sanofi R&D, Translational Disease Modeling, 65926 Frankfurt am Main, Germany

*** Correspondence:** doug.chung@certara.com

## Table S1. Summary table of IBD clinical scores that are frequently used to evaluate the disease state.

| Indication | Clinical score | Evaluation criteria | Score interpretation | Reference |
| --- | --- | --- | --- | --- |
| CD | CDAI | - Number of liquid or very soft stools (weight factor: 2) - Abdominal pain score in one week [rating,0-3] (weight factor: 5) - General well-being [rating, 1-4](weight factor: 7) - Sum of physical findings per week (weight factor: 20)   - Arthritis/arthralgia   - Mucocutaneous lesions   - Iritis/uveitis   - Anal disease   - External fistula   - Fever over 37.8**°C** - Antidiarrheal use (weight factor: 30) - Abdominal mass[no=0, equivocal=2, yes=5](weight factor: 10) - 47 minus hematocrit (males) or 42 minus hematocrit(females)(weight factor: 6) - 1-x[1-bodyweight divided by a standard weight] (weight factor: 1) | - <150 : no disease - [150-220): mild - [220-450): moderate - >450: severe | (Freeman, 2008; Kishi et al., 2022) |
| CD | CDEIS | - Evaluate the following in 5 locations: rectum, sigmoid & left colon, transverse colon, right colon and ileum - Deep ulcerations – 12 if present, 0 if absent - Superficial ulcerations – 6 if present, 0 if absent - Surface involved by disease(per 10cm)- score 0 to 10 according to length in cm - Ulcerated surface (per 10cm)- score 0 to 10 according to length in cm - Presence of ulcerated stenosis – 3 | - <3: Inactive disease - 3-8: Mild disease - 9-12: Moderate disease - ≥ 12: Severe disease | (*Info CDEIS \| Crohn’s Disease Index of Severity*, n.d.; Kishi et al., 2022; Walsh et al., 2014) |
| UC | Mayo | - Stool frequency   - 0= Normal   - 1= 1-2 stools/day more than normal   - 2= 3-4 stools/day more than normal   - 3= >4 stools/day more than normal - Rectal bleeding   - 0= None   - 1= Visible blood with stool less than half the time   - 2= Visible blood with stool half of the time or more   - 3 = Passing blood alone - Mucosal appearance in endoscopy   - 0= Normal or inactive disease   - 1= Mild disease (erythema, decreased vascular pattern, mild friability)   - 2= Moderate disease (marked erythema, absent vascular pattern, friability, erosions)   - 3= Severe disease (spontaneous bleeding, ulceration) - Physician rating of disease activity   - 0= Normal   - 1= Mild   - 2= Moderate   - 3= Severe | - 0-2: Remission - 3-5: Mild disease - 6-10: Moderate disease - 11-12: Severe disease | (Kishi et al., 2022; Lewis et al., 2008) |
| UC | MES | - Score 1: erythema, decreased vascular pattern, mild friability - Score 2: marked erythema, lack of vascular pattern, friability, erosions - Score 3: spontaneous bleeding, large ulcerations | - 0: normal mucosa - 1: Mild disease - 2: Moderate disease - 3: Severe disease | (*Info MAYO \| Partial*, n.d.) |

## Supplementary reference

Freeman, H. J. (2008). Use of the Crohn’s disease activity index in clinical trials of biological agents. *World Journal of Gastroenterology : WJG*, *14*(26), 4127. https://doi.org/10.3748/WJG.14.4127

*Info CDEIS | Crohn’s disease index of severity*. (n.d.). Retrieved October 31, 2024, from https://www.igibdscores.it/en/info-cdeis.php

*Info MAYO | Partial*. (n.d.). Retrieved October 31, 2024, from https://www.igibdscores.it/en/info-mayo-endoscopic.php

Kishi, M., Hirai, F., Takatsu, N., Hisabe, T., Takada, Y., Beppu, T., Takeuchi, K., Naganuma, M., Ohtsuka, K., Watanabe, K., Matsumoto, T., Esaki, M., Koganei, K., Sugita, A., Hata, K., Futami, K., Ajioka, Y., Tanabe, H., Iwashita, A., … Hisamatsu, T. (2022). A review on the current status and definitions of activity indices in inflammatory bowel disease: how to use indices for precise evaluation. *Journal of Gastroenterology*, *57*(4), 246–266. https://doi.org/10.1007/S00535-022-01862-Y

Lewis, J. D., Chuai, S., Nessel, L., Lichtenstein, G. R., Aberra, F. N., & Ellenberg, J. H. (2008). Use of the Non-invasive Components of the Mayo Score to Assess Clinical Response in Ulcerative Colitis. *Inflammatory Bowel Diseases*, *14*(12), 1660. https://doi.org/10.1002/IBD.20520

Walsh, A., Palmer, R., & Travis, S. (2014). Mucosal healing as a target of therapy for colonic inflammatory bowel disease and methods to score disease activity. *Gastrointestinal Endoscopy Clinics of North America*, *24*(3), 367–378. https://doi.org/10.1016/J.GIEC.2014.03.005

## Table S2. Sensitivity and specificity metrics of MES prediction model

| **MES model** | | |
| --- | --- | --- |
|  | **Sensitivity** | **Specificity** |
| **MES 0** | 0.759615384615385 | 0.886597938144330 |
| **MES 1** | 0.765517241379310 | 0.732984293193717 |
| **MES 2** | 0.442622950819672 | 0.903225806451613 |
| **MES 3** | 0.723404255319149 | 0.960176991150443 |

## Table S3. Sensitivity and specificity metrics of Mayo prediction model

| **Mayo model** | | |
| --- | --- | --- |
|  | **Sensitivity** | **Specificity** |
| **[0-2]** | 0.627659574468085 | 0.910204081632653 |
| **[3-5]** | 0.707142857142857 | 0.740890688259109 |
| **[6-10]** | 0.660550458715596 | 0.897435897435898 |
| **[11-12]** | 0.928571428571429 | 0.970464135021097 |

## Table S4. Sensitivity and specificity metrics of CDAI prediction model

| **CDAI model** | | |
| --- | --- | --- |
|  | **Sensitivity** | **Specificity** |
| **<150** | 0.832214765100671 | 0.936329588014981 |
| **150-220** | 0.837837837837838 | 0.816993464052288 |
| **220-450** | 0.736842105263158 | 0.921933085501859 |
